# Real-World Effectiveness and Noninferiority Evaluation and Comparison of Messenger RNA–Based and Protein-Based COVID-19 Vaccines: Protocol for the BEEHIVE Randomized Study With a Hybrid Effectiveness Design

**DOI:** 10.2196/80858

**Published:** 2026-01-27

**Authors:** Sarang K Yoon, German L Ellsworth, Steph Battan-Wraith, Andrew L Phillips, Rebecca V Fink, Joshua Griffin, Elizabeth A K Rowley, Jacob McKell, Ashley S Smith, Riley Campbell, Jesse Williams, Sarah W Ball, Hongwei Zhao, Brandy Warren, Matthew D Rousculp, Matthew S Thiese

**Affiliations:** 1 Division of Occupational and Environmental Health School of Medicine University of Utah Salt Lake City, UT United States; 2 Rocky Mountain Center for Occupational and Environmental Health University of Utah Salt Lake City, UT United States; 3 Westat (United States) Rockville, MD United States; 4 The Rocky Mountain Center for Occupational and Environmental Health School of Medicine University of Utah Salt Lake City, UT United States; 5 The Rocky Mountain Center for Occupational and Environmental Health University of Utah Salt Lake City, UT United States; 6 Real-World Evidence Novavax (United States) Gaithersburg United States

**Keywords:** protein, COVID-19, SARS-CoV-2, omicron, surveillance, vaccine effectiveness, clinical trial

## Abstract

**Background:**

Surveillance of COVID-19 vaccine effectiveness (VE) was extensive upon vaccine introduction; however, it declined after the withdrawal of pandemic status in May 2023. Continued monitoring of updated vaccine formulations is needed to ensure the maintenance of VE in the face of evolving viral strains.

**Objective:**

The Booster Epidemiological Evaluation of Health, Illness and Vaccine Efficacy (BEEHIVE) study (NCT06065176), a randomized trial with a hybrid design, was developed to assess the real-world VE of the 2023-2024 Pfizer–BioNTech and Novavax COVID-19 vaccine formulations targeting the XBB.1.5 SARS-CoV-2 variant.

**Methods:**

This study was designed to enroll approximately 1500 participants aged ≥18 years from the Salt Lake City, Utah, area who had previously received ≥2 doses of an authorized messenger RNA (mRNA)–based COVID-19 vaccine but had not received a dose of the 2023-2024 formulation. The study used a randomized, hybrid design comprising 2 blinded groups assigned to receive the 2023-2024 formula of either the Novavax COVID-19 vaccine or the Pfizer–BioNTech COVID-19 vaccine and a nonrandomized, observational control group of volunteers who chose not to receive a 2023-2024 vaccine dose during the study. Follow-up lasted 24 weeks and included symptom surveys and self-administered COVID-19 antigen testing, both occurring weekly. The primary aim was to compare VE (defined as prevention of symptomatic SARS-CoV-2 infection) between study-vaccinated participants and the control group. The secondary aim was to determine the relative VE of the Pfizer–BioNTech mRNA and Novavax 2023-2024 COVID-19 vaccines. Secondary objectives included assessing how the number of previous COVID-19 vaccinations impacted VE of the 2023-2024 COVID-19 vaccines; identifying predictors and associated factors for asymptomatic versus symptomatic infection and/or prolonged or severe illness; examining factors associated with post–COVID-19 conditions; and evaluating participants’ knowledge, attitudes, and practices related to COVID-19 vaccination. Participant engagement was maintained via online and text-based reminders and surveys, as well as researcher follow-up.

**Results:**

Participants were recruited from November 2023 through March 2024, with 452 and 457 participants randomized to the Novavax and Pfizer–BioNTech vaccine groups, respectively, and 279 participants enrolled in the control group. SARS-CoV-2 variants from the XBB, JN.1, KP.2, and KP.3 lineages were in circulation in the United States and the Utah region during data collection. The study ended on September 9, 2024, with results expected to be published in 2026.

**Conclusions:**

Data from this study will provide valuable real-world VE data for a dose of the Novavax COVID-19 vaccine or the Pfizer–BioNTech COVID-19 vaccine after an mRNA-based COVID-19 primary series.

**Trial Registration:**

ClinicalTrials.gov NCT06065176; https://www.clinicaltrials.gov/study/NCT06065176

**International Registered Report Identifier (IRRID):**

RR1-10.2196/80858

## Introduction

### Background

Early in the COVID-19 pandemic, there was vigilant surveillance by regulatory authorities to monitor SARS-CoV-2 cases and assess the impact of COVID-19 vaccines on controlling viral spread. When the public health emergency status was not renewed in May 2023, surveillance mechanisms greatly diminished or stopped entirely [1]. With the continued evolution of immune-evasive variants, COVID-19 remains a public health concern [1]. Maintaining surveillance of the vaccine effectiveness (VE) of updated COVID-19 vaccines targeting new strains is informative for ongoing vaccination campaigns and for the development of new vaccine formulations [2].

There are 3 COVID-19 vaccines approved for use by the US Food and Drug Administration (FDA) [3-5]. Of these, 2 are messenger RNA (mRNA)–based vaccines (Pfizer–BioNTech COVID-19 vaccine and Moderna COVID-19 vaccine) and 1 is an adjuvanted recombinant spike protein–based vaccine (Novavax COVID-19 vaccine). A key difference among these formulations is the use of mRNA encoding the SARS-CoV-2 spike protein in the Pfizer–BioNTech and Moderna vaccines [3,4], compared with the use of recombinant spike protein with Matrix-M adjuvant in the protein-based Novavax vaccine [5-7]. Prototype monovalent COVID-19 vaccines of each of these formulations, targeting the ancestral Wuhan strain, have undergone a series of updates since their initial authorization (ie, bivalent Wuhan and BA.4/5, monovalent XBB.1.5, and monovalent JN.1 lineage) to target circulating lineages of relevance at the time [8].

There are limited clinical and real-world data on the VE of XBB.1.5–based vaccines [9,10]. Notably, existing reports focus on assessments of symptomatic infection over a limited period. A better understanding is needed of the protection these vaccine formulations may provide against both symptomatic and asymptomatic infection for several months after vaccination.

### Objectives

The Booster Epidemiological Evaluation of Health, Illness and Vaccine Efficacy (BEEHIVE) study (NCT06065176) was developed as a randomized trial with a hybrid design that includes a randomized comparison between active vaccine groups and an observational control group that did not receive a dose of the study vaccine. The goal was to assess the VE of the 2023-2024 formulations (against XBB.1.5) for the Pfizer–BioNTech and Novavax COVID-19 vaccines in a real-world setting [11]. Importantly, this study was designed with a comprehensive surveillance structure to identify SARS-CoV-2 infections, regardless of symptom presence, over a 6-month period following study vaccination. The study protocol and end points are described here, along with the novel recruitment methods and strategies used to maintain participant engagement.

## Methods

### Study Design

The BEEHIVE study was designed to enroll approximately 1500 participants. Participants were aged ≥18 years; resided in Salt Lake City, Utah (including a 60-mile radius of surrounding areas); had previously received ≥2 doses of an mRNA-based COVID-19 vaccine authorized by the US FDA; and met the eligibility criteria described in Textbox 1.

**Inclusion and exclusion criteria.**

**Inclusion criteria**

- Aged ≥18 years
- Previous receipt of ≥2 doses of a US Food and Drug Administration (FDA)–authorized messenger RNA–based COVID-19 vaccine
- Comfortable reading and responding to SMS text messaging and emails sent in English (on own or with interpreter assistance)
- Plan to remain in the greater Salt Lake City, Utah, area for the next 12 months
- Daily access to the internet (via cell phone, laptop, desktop, or tablet) and a phone with text messaging capabilities
- Willingness to complete the following:
  Weekly symptom and illness surveillance (sent via text and email)
  Enrollment, midstudy, and end-of-study surveys
- Willingness to be contacted periodically by study staff via text, email, and/or telephone as part of study activities
- Willingness to self-collect rapid antigen tests (approved by FDA Emergency Use Authorization for COVID-19 detection) weekly, when prompted for study purposes, if experiencing a qualifying symptomatic illness, or upon rapid antigen test confirmation of an asymptomatic infection and to report the results via the study portal
- Willingness to attend an in-person visit to receive a supply of rapid antigen tests and training on their use (all participants) and to receive an updated 2023-2024 COVID-19 vaccine (randomized participants)

**Exclusion criteria**

- Lives with another person who is already enrolled in the Booster Epidemiological Evaluation of Health, Illness and Vaccine Efficacy (BEEHIVE) study, as reported on the eligibility survey
- Previous hypersensitivity reaction to a COVID-19 vaccine, as reported on the eligibility survey
- COVID-19 infection (infection confirmed via self-report of a positive real-time reverse transcription polymerase chain reaction assay and/or rapid antigen test) ≤90 days before enrollment
- Receipt of a COVID-19 vaccine ≤90 days before enrollment
- Participation in other vaccine or investigational product trials
- Medical history of immunosuppression
- Receipt of Johnson & Johnson COVID-19 vaccine before study enrollment
- Receipt of any investigational prevention therapies for SARS-CoV-2 infections, such as prophylactic antiviral medications or other immune system–modifying interventions, ≤90 days before trial vaccine administration
- Unwillingness to provide electronic consent or to self-report occupation, work responsibilities, or previous COVID-19 illness

**Figure 1:**
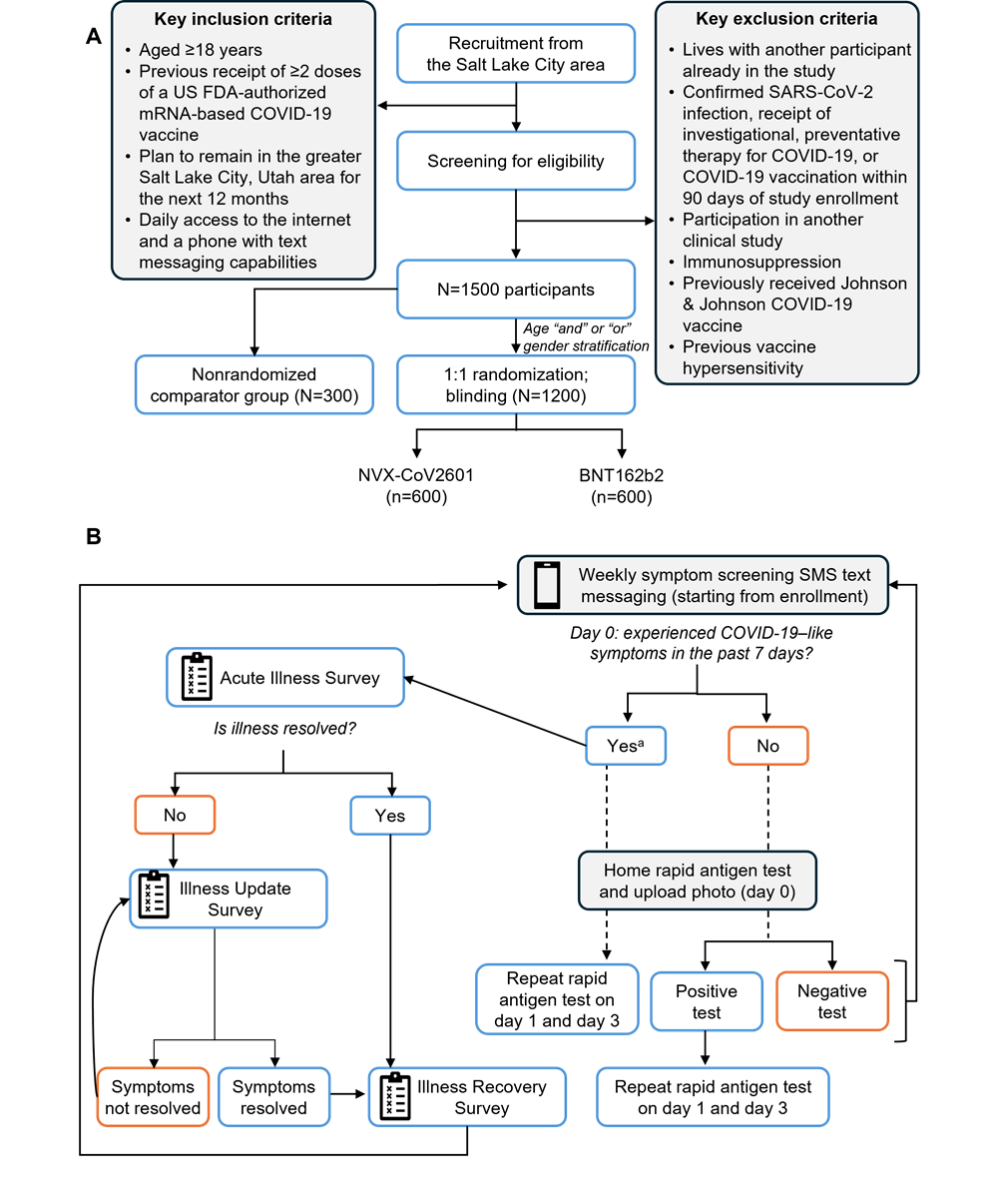
Study design: (A) recruitment, screening, and study schema, and (B) decision tree for symptom and rapid antigen test monitoring. For this study, the interpretation of results is based on the participant’s status (eg, asymptomatic or has COVID-19–like illness symptoms) on the first day (day 0) of each testing. Additional tests on days 1 and 3 were required if a participant had symptoms on day 0 and if a test was positive on day 0, regardless of symptoms status. To be considered a true negative case, all 3 tests must result as negative. FDA: Food and Drug Administration; mRNA: messenger RNA.

Study groups comprised 2 randomized, blinded groups who received the 2023-2024 formulation of either the Novavax COVID-19 vaccine or the Pfizer–BioNTech COVID-19 vaccine and a nonrandomized, observational control group of volunteers who participated in the study but did not elect to receive a study vaccine or any 2023-2024 COVID-19 vaccine formulation during the surveillance period (Figure 1A).

### Participant Recruitment

Several unique mechanisms were used to facilitate participant recruitment. Radio and television advertisements were deployed in the Salt Lake City area, and flyers for the BEEHIVE study were distributed to patient groups representative of populations that could meet eligibility criteria (Multimedia Appendix 1). Participants from previous COVID-19 studies (ie, RECOVER [Research on the Epidemiology of SARS-CoV-2 in Essential Response Personnel] and PROTECT [Pediatric Research Observing Trends and Exposures in COVID-19 Timelines]) [12,13] or an influenza vaccine study (ie, RAIVEN [Randomized Assessment of Influenza Vaccine Efficacy Network]) [14] who consented to be contacted for future studies were sent recruitment invitations.

Additional outreach efforts included joining occupational health vaccination campaigns and other existing mass-vaccination events, advertising through social media and local news outlets, staffing booths at community events, and deploying mobile vaccination stations. Collaborations were established with local community groups, clinics, businesses, universities, older adult centers, and fire and police departments. Potential participants received a link to the online eligibility survey via email and were informed of their eligibility status upon survey completion. After completion of consent and Health Insurance Portability and Accountability Act (HIPAA) authorization forms, eligible participants were invited to create an online portal account, complete the enrollment survey, and schedule an in-person enrollment visit online (Multimedia Appendix 2).

### Vaccination History

For participants who provided consent to having their vaccine records obtained by the study staff, the state immunization information system or registry was accessed to obtain COVID-19 and influenza vaccination status and history. Participants were also asked to self-report their COVID-19 and influenza vaccination history and to provide vaccine documentation to the study.

### Study Objectives

There were 2 aims of the BEEHIVE study. The primary aim was to compare VE between study-vaccinated participants and a control group who declined receipt of an updated COVID‑19 vaccine (2023-2024 formula) in preventing symptomatic SARS-CoV-2 infection. The secondary aim was to determine the relative VE (rVE) of the 2023-2024 formulation of the Pfizer–BioNTech mRNA and Novavax COVID-19 vaccines in preventing symptomatic SARS-CoV-2 infection.

Secondary objectives included evaluating the effect of the number of previous COVID-19 vaccinations on the rVE of the Novavax COVID-19 vaccine. Additional secondary objectives were to assess predictors of asymptomatic versus symptomatic SARS-CoV-2 infection; clinical characteristics and outcomes associated with COVID-19, including illness duration and severity; sociodemographic and health characteristics associated with prolonged or severe illness; the impact of COVID-19 on indicators of functioning (eg, missed work, ability to complete normal work and home activities, and working while ill); the proportion of COVID-19 illnesses that were medically attended; and factors associated with seeking medical care and treatment. Illness characteristics and duration for primary infection versus reinfection will be compared, and the effect modification of VE by sociodemographic characteristics, occupation, health status, and other risk factors will be evaluated.

The goals of additional outcomes analyses are to assess whether the 2023-2024 vaccine formulation modifies illness severity and duration among participants with breakthrough infection. Data related to the incidence of post–COVID-19 condition (PCCs) were collected, along with identification of factors associated with PCC symptoms; assessment of whether these symptoms differed between randomized and control study groups; evaluation of whether PCC symptoms were modified by the number of previous vaccine doses; and assessment of the impact of PCC symptoms on overall functioning and mental health. PCCs were defined as any self-reported condition (continuous and relapsing or remitting) occurring after COVID-19 and persisting for at least 1 month after the initial illness, including fatigue, memory problems, dyspnea, sleep problems, joint pain, and/or newly developed medical conditions (eg, myocarditis, heart failure, cardiac dysrhythmias, stroke, chronic kidney diseases, neurologic conditions, and diabetes) [15]. PCCs were grouped by symptom category (eg, cardiovascular, respiratory, and neurological) and by number of symptoms (≥1 or ≥2). Finally, the knowledge, attitudes, and practices (KAPs) of participants related to 2023-2024 vaccines are to be characterized, and associations between KAPs and subsequent vaccination behaviors (including vaccine refusal, hesitancy, or incomplete adherence to vaccination recommendations) will be examined.

### Ethical Oversight

Before study implementation, the protocol, informed consent form, participant education and recruitment materials, data collection instruments, and other protocol-related documents were approved by the University of Utah and Westat institutional review boards (I00172738 and 8084.01, respectively). In-person signatures were obtained for informed consent and HIPAA authorization forms. Participants were able to access copies of the consent forms through their portal accounts and received a signed hard copy. All participants provided consent and were allowed to withdraw at any time.

The research data platform and study database were maintained by Westat, Inc. To protect confidentiality, we used assigned identification numbers rather than personal information on study forms. We stored data in locked files and/or secured computers. For information from this study presented publicly or published in a medical journal, individuals will not be identified by name or by any other personally identifiable information. Participants received compensation for study activities (refer to the Remuneration and Engagement section for additional details).

### Randomization and Blinding

A computerized random-number generator produced the randomized list for the active vaccinated arms before study enrollment. Randomly permuted blocks (designated A-F) of size 6, with 3 assignments to each vaccine type per block, were generated to achieve 1:1 allocation. Randomization was stratified by sex and age (18-49 years and ≥50 years). Eligible participants who provided consent indicating agreement to receive an updated 2023-2024 vaccine were able to schedule their first study visit and vaccination appointment.

Participants were randomized to either the Novavax COVID-19 vaccine group or the Pfizer–BioNTech COVID-19 vaccine group; however, based on appointment selection and attendance, some participants who consented may not have completed randomization or received a study vaccine. Enrolled participants who provided consent to the study but declined receipt of the 2023-2024 formulation were included in the nonrandomized control group until enrollment was full. Participants who changed their minds at the time of their first study visit were able to switch groups (ie, initially agreed to be randomized to receive a vaccine but then switched to the nonrandomized control group, or vice versa) at the time of their appointment.

Randomized participants, study investigators, and study personnel were blinded to the type of vaccine received during the study, and multiple procedures were performed to maintain blinding. Specifically, vaccine packaging, administration, and documentation practices were standardized to avoid any distinguishable characteristics between the 2 vaccines. Additionally, all study staff, except for licensed health care providers who administered the vaccines, were blinded and received training on the importance of consistent data collection and maintaining blinding. Vaccines were prepared in a designated space that was obscured from participants and study personnel.

Study personnel who administered the vaccines were unblinded and underwent specialized training to ensure that vaccine type was not inadvertently disclosed. These personnel were not involved in data collection or analysis and interacted with the participants only to confirm their identity and administer the vaccine. The Westat randomization coordinator and limited data management staff were also unblinded to facilitate development and maintenance of the randomization list and systems. These staff were not involved in data collection, data analysis, or direct interaction with participants. Participants and study investigators were unblinded at the end of data collection and at the conclusion of the study, respectively. All study-related documents and samples contain a unique identifier for each participant to preserve blinding.

### Interventions

Randomized participants were to receive a single dose of the study vaccine (0.3 ml for Pfizer–BioNTech or 0.5 ml for Novavax) administered in the deltoid muscle and were to be monitored for 15 minutes after vaccination for any allergic reactions. An investigational new drug application was submitted to and approved by the US FDA for use of the Novavax COVID-19 vaccine in this study, as it was authorized under an Emergency Use Authorization at the time of the study [5].

### Surveillance: Surveys

Surveys were developed based on a pre-existing framework used in prospective clinical trials evaluating influenza and COVID-19 vaccines [13,14,16]. In the enrollment survey, the participants self-reported their sociodemographic characteristics, health status and behaviors, occupational status and employment history, chronic medical conditions, COVID-19 and influenza vaccination history, and previous SARS-CoV-2 infections.

Postvaccination surveys were shared 24 hours, 48 hours, and 6 days after study vaccination to obtain information on potential vaccine side effects. Active surveillance of outcomes occurred for participants on the same day each week, beginning the week following the enrollment and study vaccination visit, and continued for 24 weeks (Figure 1B).

Symptoms were assessed weekly through surveillance and illness messaging surveys for 24 weeks after enrollment and study vaccination. Surveillance included weekly subjective assessment of COVID-19–like illness (CLI), which was defined as experiencing at least 1 of the following symptoms: chills, malaise, fatigue, headache, cough, shortness of breath, sore throat, runny nose or nasal congestion, nausea or vomiting, diarrhea, muscle or body aches, or change in smell or taste.

Follow-up surveys were deployed at designated time points and allowed participants to provide sociodemographic and illness updates, assess KAPs, and collect information on PCCs (Textbox 2).

**Focus areas of follow-up surveys.**

- Update information on occupation and work responsibilities; health status; potential exposures to COVID-19 at work, home, and in the community; and use of personal protective equipment in these settings
- Report a qualifying illness and/or submit illness specimens for any reason and provide an estimate of when the illness event occurred
- Assess participants’ knowledge, attitudes, and practices regarding COVID-19 vaccines; intention to be vaccinated; and, after vaccination, recall of overall health status on the days following vaccination
- Report symptoms related to post–COVID-19 conditions (eg, symptoms lasting longer than 1 month), if applicable

During the surveillance period, if a participant indicated a CLI symptom on a weekly survey or reported an acute illness between their designated weekly surveillance contacts, they were directed to the Acute Illness Survey (Figure 1B). In this survey, they were asked to identify qualifying symptoms from a detailed checklist, confirm the date of symptom onset, and indicate whether the symptoms were ongoing. If the participant did not confirm symptoms of CLI, they returned to weekly surveillance. If the symptoms were ongoing, participants were prompted to collect a specimen and submit the results according to study procedures. Illness Update and Illness Recovery Surveys were deployed to monitor symptoms until a participant reported ≥90% recovery (Figure 1B). Survey data were collected electronically via a text messaging interface and online surveys (Multimedia Appendix 2).

### Surveillance: Testing

All participants received OHC COVID-19 Antigen Self-Test kits (Osang Healthcare) for weekly use, along with instructions on how to use the kit, interpret results, and upload results to the study portal (Multimedia Appendix 1). Assay sensitivity is 91% and relative specificity is 99%, as described by the manufacturing company. Anterior nasal swab specimens were self-collected weekly on the same day (day 0), and a photograph of the results was uploaded regardless of symptom status (Figure 1B).

If a participant reported an acute illness and/or collected an illness specimen on or outside their assigned routine specimen day, they were asked to collect a second specimen on the next day (day 1) and a third specimen 2 days later (day 3). For this study, the interpretation of test results was based on the participant’s symptom status on the first day of testing (day 0) and whether they did or did not have CLI symptoms.

### Safety Assessments

Safety assessments included monitoring and recording of solicited (local and systemic reactogenicity events) and unsolicited adverse events (AEs), serious AEs, and AEs of special interest (AESIs). Solicited AEs were collected through the Postvaccination Survey. Unsolicited AEs could be reported at any time from study vaccination through completion of the last study-related procedure.

The relation of each event to the study vaccine (related or not related), whether the event was expected or unexpected, whether the event was serious or not, and the intensity of AEs and serious AEs were investigator determined. Severity was categorized as mild (grade 1), moderate (grade 2), severe (grade 3), life-threatening (grade 4), or death (grade 5). Protocol-defined AESIs were myocarditis and pericarditis and were collected by participant report, with verification through medical record review and follow-up interviews by study clinicians to obtain additional details.

Participants received an informational sheet describing symptoms of myocarditis and pericarditis, a list of nearby medical facilities for care seeking, and a letter for their evaluating physician, which included recommendations for clinical management. Probable and confirmed cases of AESIs were defined as previously described [17]. Safety reporting related to the investigational new drug status of the Novavax COVID-19 vaccine included sharing suspected unexpected serious adverse reactions and relevant medication errors with the appropriate regulatory authorities.

### Statistical Analyses

The primary population for VE analyses will be the modified intent-to-treat (mITT) population, which includes all randomized participants who received a study vaccine and responded to ≥1 active surveillance survey, and all participants in the control group. Any participant with a CLI-associated SARS-CoV-2 infection confirmed by COVID-19 rapid antigen test 0 to 6 days after vaccination will be excluded from the mITT population. Analyses will also be performed with the per-protocol population, which includes participants who met eligibility criteria, received a study vaccine per protocol, participated in study surveillance by responding to at least 1 surveillance contact during the SARS-CoV-2 circulation period (active surveillance), and did not receive another SARS-CoV-2 vaccine outside of the study during the study period. If participants were in the nonvaccinated control group but subsequently received a 2023-2024 updated vaccine, they will be censored at that point. Any participants withdrawing early from the study will be censored at that time and included in analyses. There will be no imputation for missing data.

Using publicly available data [18] on COVID-19 cases in Utah from October 1, 2022, to May 28, 2023, the average rate of reported cases in fully vaccinated individuals from fall 2022 through spring 2023 was 39.64 per 100,000 person-days. On the basis of previously reported survey results of clinic and laboratory testing rates for COVID-19 [19] and the use of at-home test kits in this study, the estimated case reporting was 43.1% higher. Using these values and adjustments to the observed rates of infection in Utah in fall 2022 leads to a conservative estimate of the rate of CLI-associated SARS-CoV-2 infection in the control group, confirmed by COVID-19 rapid antigen test, of 80 per 100,000 person-days. With 1500 participants enrolled (1200 randomized and 300 nonrandomized control), assuming ≤15% attrition, there would have been 80% power to detect a VE of ≥49.4% for the primary aim (ie, VE between randomized and control participant groups), examined with a 2-sided alpha 0.05 superiority test.

For the secondary aim, noninferiority of the Novavax COVID-19 vaccine versus the Pfizer–BioNTech COVID-19 vaccine will be declared if the lower one-sided 95% CI for rVE is >50%. With 600 participants per vaccine group and an assumed infection rate of 60 per 100,000 person-days in the Pfizer–BioNTech COVID-19 vaccine arm and a rVE of 10% for the Novavax COVID-19 vaccine versus the Pfizer–BioNTech COVID-19 vaccine (ie, Novavax>Pfizer–BioNTech) [20,21], there would be 80% power for the noninferiority objective. The positive rVE used in the sample size calculation is based on estimates from large retrospective studies [20,21], which showed that the Novavax COVID-19 vaccine provides added protection against medically attended and severe SARS-CoV-2 infection. Sample size calculations for study aims were performed in PASS 2023 (NCSS, LLC) using the Cox proportional hazards model to estimate hazard ratios. Certain secondary objectives will be underpowered if there are insufficient numbers of nonsymptomatic cases, reinfections, and/or PCCs during the study period.

Participant demographics and characteristics will be summarized descriptively in the mITT population. The Cox proportional hazards model will be used to analyze time-to-event data to evaluate VE in preventing symptomatic COVID-19 infection. The observation period started 7 days after the receipt of the updated 2023-2024 vaccine for the randomized groups and at the enrollment date for the control group. Event onset time is defined as the earlier of the date of a positive rapid antigen test or the date of CLI symptom onset reported within a week of a positive rapid antigen test. Covariates of age (continuous variable) and vaccination dose history (categorical variable) will be included in the model to control for confounding. Additional demographic variables will be considered for inclusion if they change the hazard ratio for the treatment variable by ≥10%. Subgroup analyses based on age group and immune challenge (ie, most recent COVID-19 infection or vaccination) were conducted to assess potential effect modification across age and immune-challenge subgroups. All analyses will be performed using SAS (version 9.4 or higher; SAS Institute Inc), or R (version 4.1.2 or higher; R Foundation for Statistical Computing).

### Remuneration and Engagement

Participants received compensation in the form of Amazon, Inc. gift cards, with the value proportional to the number of study activities completed (Multimedia Appendix 1). Newsletters were shared throughout the study with investigators and participants, including information such as enrollment numbers, instructions on how to remain compliant and up-to-date with study protocol and surveys, and updates on COVID-19 activity in the area. Study branding was consistently presented from recruitment throughout the study.

## Results

### Participant Enrollment and Data Collection

Participant screening occurred from November 17, 2023, through March 14, 2024. Monitoring occurred from enrollment through the end of the study on September 9, 2024. A total of 1188 participants were enrolled: 909 in the randomized vaccine groups (Novavax COVID-19 vaccine, 452/909, 49.7%; Pfizer–BioNTech COVID-19 vaccine, n=457, 50.3%) and 279 in the nonrandomized control group. Data analyses are ongoing, and results are expected to be published in 2026.

### Vaccination Timing and SARS-CoV-2 Circulation

**Figure 2:**
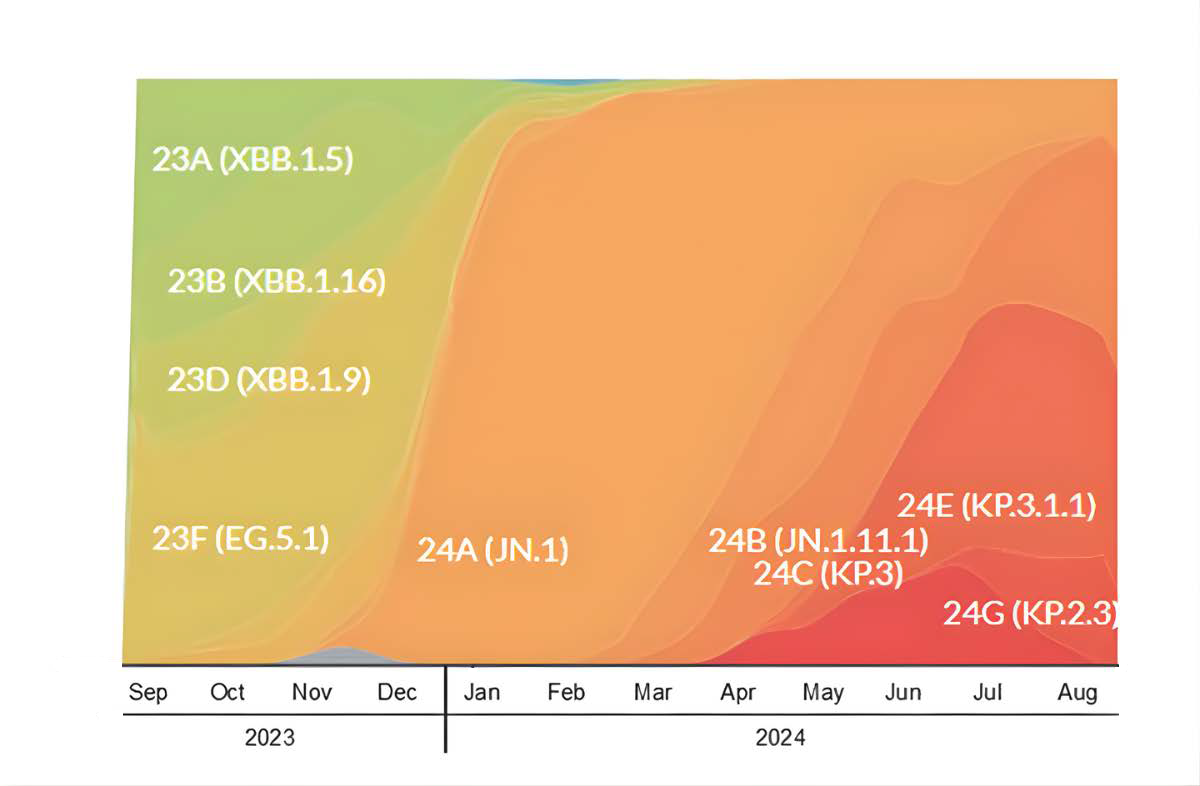
Circulating SARS-CoV-2 strains during the Booster Epidemiological Evaluation of Health, Illness and Vaccine Efficacy (BEEHIVE) study [22] (adapted from nexstrain.org under the CC-BY-4.0 license).

Vaccination and data collection during the study occurred when XBB.1.5-, JN.1-, KP.2-, and KP.3-lineage SARS-CoV-2 variants were circulating in the United States and the Utah region (Figure 2 [22]). Study vaccinations were relatively evenly distributed throughout the enrollment period, with most occurring when XBB.1.5 and JN.1 represented ≥50% of SARS-CoV-2 strains circulating in Utah (January 19, 2023, and December 30, 2023, respectively).

## Discussion

The BEEHIVE study will provide real-world VE data for the Novavax COVID-19 vaccine when administered as an additional dose after at least a primary series of an mRNA-based COVID-19 vaccine and will compare these VE data with those of the Pfizer–BioNTech COVID-19 mRNA vaccine. Outcomes may provide information on the benefits of heterologous dosing or the use of mixed vaccine formulations, for administration beyond primary vaccination.

Access to participant cohorts from previous studies allowed recruitment of individuals who were more likely to participate and facilitated access to sociodemographic information and vaccination history. Mobile vaccine clinics, attendance at community events, and collaborations with civic groups were more effective in engaging typically underrepresented communities (eg, older adults and racial and ethnic minority individuals) [23,24] than standard clinical trial site strategies. Electronic surveillance and the use of online and text-based surveys supported efficient data collection and sustained participant engagement throughout the 24-week surveillance period.

A potential limitation of this study includes the underrepresentation of participants in the older adult categories. Additionally, the variability in SARS-CoV-2 circulation and the introduction of new variant strains during the study period would impact infection rates and symptom severity. Notably, the testing kits, which were identical for all study participants, had received Emergency Use Authorization from the US FDA for Omicron and subvariant testing. The single-site nature of the trial may restrict geographic diversity among the participants; however, the feasibility of a real-world study design was established and can be adapted for future multisite studies. Because rapid antigen tests were used as part of the real-world study design, repeat testing procedures were included in the design to mitigate concerns regarding the absence of more robust clinical assays (eg, polymerase chain reaction). Another consideration is that the control group that opted out of receiving a study vaccination may introduce selection bias. Briefly, participants who declined the recommended vaccine may have differed demographically or behaviorally from those who chose to receive a study vaccine [25].

Participant enrollment (n=1188) was lower than the target of 1500 described in sample size calculations. We acknowledge that the study is underpowered due to recruitment challenges, and the implications of the smaller sample size on effect detectability will be discussed in the final report. Additionally, assumptions of superior rVE of Novavax compared with mRNA vaccines based on medically attended COVID-19 infection may not directly translate to protection against symptomatic SARS-CoV-2 infection. Future noninferiority studies should consider sample size calculations assuming an rVE of 0 to ensure a more robust study design.

Continued surveillance of COVID-19 cases and VE data for vaccine formulations updated based on predominant SARS-CoV-2 strains is necessary to inform public health policy and management. The BEEHIVE study will provide valuable real-world VE data for populations representative of individuals who have received the 2023-2024 updated COVID-19 vaccine, including those who received a dose of the Novavax COVID-19 vaccine after receipt of ≥2 mRNA-based COVID-19 vaccines.

## Data Availability

The datasets generated or analyzed during this study are available in the ClinicalTrials.gov database [11].

## Appendix

Multimedia Appendix 1 Recruitment and engagement materials.

Multimedia Appendix 2 Electronic interfaces.

## Abbreviations

AE: adverse event
AESI: adverse event of special interest
BEEHIVE: Booster Epidemiological Evaluation of Health, Illness and Vaccine Efficacy
CLI: COVID-19–like illness
FDA: Food and Drug Administration
HIPAA: Health Insurance Portability and Accountability Act
KAP: knowledge, attitudes, and practices
mITT: modified intent-to-treat
mRNA: messenger RNA
PCC: post–COVID-19 condition
rVE: relative vaccine effectiveness
VE: vaccine effectiveness

## References

[1] Silk BJ, Scobie HM, Duck WM, Palmer T, Ahmad FB, Binder AM, Cisewski JA, Kroop S, Soetebier K, Park M, Kite-Powell A, Cool A, Connelly E, Dietz S, Kirby AE, Hartnett K, Johnston J, Khan D, Stokley S, Paden CR, Sheppard M, Sutton P, Razzaghi H, Anderson RN, Thornburg N, Meyer S, Womack C, Weakland AP, McMorrow M, Broeker LR, Winn A, Hall AJ, Jackson B, Mahon BE, Ritchey MD (2023). COVID-19 surveillance after expiration of the public health emergency declaration - United States, May 11, 2023. MMWR Morb Mortal Wkly Rep.

[2] Recommendation for the 2023-2024 formula of COVID-19 vaccines in the U.S. US Food and Drug Administration.

[3] (2024). Spikevax (COVID-19 vaccine, mRNA); 2024-2025 formula. Moderna, Inc.

[4] (2024). Comirnaty (COVID-19 vaccine, mRNA); 2024-2025 formula. Pfizer–BioNTech.

[5] Package Insert and Patient Package Insert - NUVAXOVID. US Food and Drug Administration.

[6] Reimer JM, Karlsson KH, Lövgren-Bengtsson K, Magnusson SE, Fuentes A, Stertman L (2012). Matrix-M™ adjuvant induces local recruitment, activation and maturation of central immune cells in absence of antigen. PLoS One.

[7] Stertman L, Palm AE, Zarnegar B, Carow B, Lunderius Andersson C, Magnusson SE, Carnrot C, Shinde V, Smith G, Glenn G, Fries L, Lövgren Bengtsson K (2023). The Matrix-M™ adjuvant: a critical component of vaccines for the 21 century. Hum Vaccin Immunother.

[8] Katella K (2025). Comparing the COVID-19 vaccines: how are they different?. Yale Medicine.

[9] Lin DY, Du Y, Xu Y, Paritala S, Donahue M, Maloney P (2024). Effectiveness of XBB.1.5 vaccines against Omicron subvariants. Med Res Arch.

[10] Link-Gelles R, Ciesla AA, Mak J, Miller JD, Silk BJ, Lambrou AS, Paden CR, Shirk P, Britton A, Smith ZR, Fleming-Dutra KE (2024). Early estimates of updated 2023-2024 (Monovalent XBB.1.5) COVID-19 vaccine effectiveness against symptomatic SARS-CoV-2 infection attributable to co-circulating Omicron variants among immunocompetent adults - increasing community access to testing program, United States, September 2023-January 2024. MMWR Morb Mortal Wkly Rep.

[11] (2023). The Efficacy of the 2023-2024 updated COVID-19 vaccines against COVID-19 infection (BEEHIVE). National Library of Medicine.

[12] Burns J, Rivers P, LeClair LB, Jovel KS, Rai RP, Lowe AA, Edwards LJ, Khan SM, Mathenge C, Ferraris M, Kuntz JL, Lamberte JM, Hegmann KT, Odean MJ, McLeland-Wieser H, Beitel S, Odame-Bamfo L, Schaefer Solle N, Mak J, Phillips AL, Sokol BE, Hollister J, Ochoa JS, Grant L, Thiese MS, Jacoby KB, Lutrick K, Pubillones FA, Yoo YM, Rentz Hunt D, Ellingson K, Berry MC, Gerald JK, Lopez J, Gerald LB, Wesley MG, Krupp K, Herring MK, Madhivanan P, Caban-Martinez AJ, Tyner HL, Meece JK, Yoon SK, Fowlkes AL, Naleway AL, Gwynn L, Burgess JL, Thompson MG, Olsho LE, Gaglani M (2022). Pediatric Research Observing Trends and Exposures in COVID-19 Timelines (PROTECT): protocol for a multisite longitudinal cohort study. JMIR Res Protoc.

[13] Edwards LJ, Fowlkes AL, Wesley MG, Kuntz JL, Odean MJ, Caban-Martinez AJ, Dunnigan K, Phillips AL, Grant L, Herring MK, Groom HC, Respet K, Beitel S, Zunie T, Hegmann KT, Kumar A, Joseph G, Poe B, Louzado-Feliciano P, Smith ME, Thiese MS, Schaefer-Solle N, Yoo YM, Silvera CA, Mayo Lamberte J, Mak J, McDonald LC, Stuckey MJ, Kutty P, Arvay ML, Yoon SK, Tyner HL, Burgess JL, Hunt DR, Meece J, Gaglani M, Naleway AL, Thompson MG (2021). Research on the epidemiology of SARS-CoV-2 in essential response personnel (RECOVER): protocol for a multisite longitudinal cohort study. JMIR Res Protoc.

[14] Grant L, Whitaker JA, Yoon SK, Lutrick K, Bhargava SP, Brown C, Zaragoza E, Fink RV, Meece J, Wielgosz K, El Sahly H, Hegmann KT, Lowe AA, Southworth A, Tatum T, Ball SW, Levine MZ, Thiese MS, Battan-Wraith S, Barnes J, Phillips AL, Fry AM, Dawood FS, Randomized Assessment of Influenza Vaccine Efficacy Network (RAIVEN) (2024). Relative effectiveness and immunogenicity of quadrivalent recombinant influenza vaccine versus egg-based inactivated influenza vaccine among adults aged 18-64 years: results and experience from a randomized, double-blind trial. Open Forum Infect Dis.

[15] Mak J, Khan S, Britton A, Rose S, Gwynn L, Ellingson K, Meece J, Feldstein LR, Tyner H, Edwards LJ, Thiese MS, Naleway A, Gaglani M, Solle N, Burgess JL, Lamberte JM, Shea M, Hunt-Smith T, Caban-Martinez A, Porter C, Wiegand R, Rai R, Hegmann KT, Hollister J, Fowlkes A, Wesley M, Philips AL, Rivers P, Bloodworth R, Newes-Adeyi G, Olsho LE, Yoon SK, Saydah S, Lutrick K (2025). Association of messenger RNA coronavirus disease 2019 (COVID-19) vaccination and reductions in post COVID conditions following severe acute respiratory syndrome coronavirus 2 infection in a US prospective cohort of essential workers. J Infect Dis.

[16] Thompson MG, Burgess JL, Naleway AL, Tyner H, Yoon SK, Meece J, Olsho LE, Caban-Martinez AJ, Fowlkes AL, Lutrick K, Groom HC, Dunnigan K, Odean MJ, Hegmann K, Stefanski E, Edwards LJ, Schaefer-Solle N, Grant L, Ellingson K, Kuntz JL, Zunie T, Thiese MS, Ivacic L, Wesley MG, Mayo Lamberte J, Sun X, Smith ME, Phillips AL, Groover KD, Yoo YM, Gerald J, Brown RT, Herring MK, Joseph G, Beitel S, Morrill TC, Mak J, Rivers P, Poe BP, Lynch B, Zhou Y, Zhang J, Kelleher A, Li Y, Dickerson M, Hanson E, Guenther K, Tong S, Bateman A, Reisdorf E, Barnes J, Azziz-Baumgartner E, Hunt DR, Arvay ML, Kutty P, Fry AM, Gaglani M (2021). Prevention and attenuation of COVID-19 with the BNT162b2 and mRNA-1273 vaccines. N Engl J Med.

[17] Gargano JW, Wallace M, Hadler SC, Langley G, Su JR, Oster ME, Broder KR, Gee J, Weintraub E, Shimabukuro T, Scobie HM, Moulia D, Markowitz LE, Wharton M, McNally VV, Romero JR, Talbot HK, Lee GM, Daley MF, Oliver SE (2021). Use of mRNA COVID-19 vaccine after reports of myocarditis among vaccine recipients: update from the advisory committee on immunization practices - United States, June 2021. MMWR Morb Mortal Wkly Rep.

[18] COVID-19 data - coronavirus dashboard. Utah Department of Health and Human Services.

[19] Luisi N, Sullivan PS, Sanchez T, Bradley H, Fahimi M, Shioda K, Nelson KN, Lopman BA, Siegler AJ (2023). Use of COVIDTests.gov at-home test kits among adults in a national household probability sample - United States, 2022. MMWR Morb Mortal Wkly Rep.

[20] Gwak E, Choe SA, Bolormaa E, Choe YJ, Wang C, Fix J, Vadivale M, Rousculp MD (2025). Relative effectiveness of homologous NVX-CoV2373 and BNT162b2 COVID-19 vaccinations in South Korea. Vaccine.

[21] Gwak E, Choe SA, Kim K, Bolormaa E, Fix J, Vadivale M, Rousculp MD, Choe YJ (2025). Real-world effectiveness of NVX-CoV2373 and BNT162b2 mRNA COVID-19 vaccination in South Korea. Vaccine.

[22] (2025). Genomic epidemiology of SARS-CoV-2 with subsampling focused on North America since pandemic start. Nextstrain.

[23] Bibbins-Domingo K, Helman A, National Academies of Sciences, Engineering, and Medicine, Policy and Global Affairs, Committee on Women in Science, Engineering, and Medicine, Committee on Improving the Representation of Women and Underrepresented Minorities in Clinical Trials and Research (2022). Why diverse representation in clinical research matters and the current state of representation within the clinical research ecosystem. Improving Representation in Clinical Trials and Research: Building Research Equity for Women and Underrepresented Groups.

[24] van Marum RJ (2020). Underrepresentation of the elderly in clinical trials, time for action. Br J Clin Pharmacol.

[25] Groom HC, Biel FM, Crane B, Sun E, Georgescu JP, Weintraub ES, McNeil MM, Jazwa A, Smith N, Owens-Jasey C, Naleway AL, Schmidt T (2024). Disparities in COVID-19 vaccination receipt by race, ethnicity, and social determinants of health among a large patient population in a network of community-based healthcare centers. Vaccine.

